# Inferring population-level contact heterogeneity from common epidemic data

**DOI:** 10.1098/rsif.2012.0578

**Published:** 2013-01-06

**Authors:** J. Conrad Stack, Shweta Bansal, V. S. Anil Kumar, Bryan Grenfell

**Affiliations:** 1 Department of Biology, Pennsylvania State University, University Park, PA 16802-5301, USA; 2 Center for Infectious Disease Dynamics, Pennsylvania State University, University Park, PA 16802-5301, USA; 3 Fogarty International Center, National Institutes of Health, Bethesda, MD 20892-220, USA; 4 Department of Computer Science and Virginia Bioinformatics Institute, Virginia Polytechnic Institute and State University, Blacksburg, VA 24061, USA; 5 Department of Ecology and Evolutionary Biology and Woodrow Wilson School, Princeton University, Princeton, NJ 08540, USA

**Keywords:** network model, infectious disease, epidemic data, statistical inference, contact heterogeneity

## Abstract

Models of infectious disease spread that incorporate contact heterogeneity through contact networks are an important tool for epidemiologists studying disease dynamics and assessing intervention strategies. One of the challenges of contact network epidemiology has been the difficulty of collecting individual and population-level data needed to develop an accurate representation of the underlying host population's contact structure. In this study, we evaluate the utility of common epidemiological measures (*R*_0_, epidemic peak size, duration and final size) for inferring the degree of heterogeneity in a population's unobserved contact structure through a Bayesian approach. We test the method using ground truth data and find that some of these epidemiological metrics are effective at classifying contact heterogeneity. The classification is also consistent across pathogen transmission probabilities, and so can be applied even when this characteristic is unknown. In particular, the reproductive number, *R*_0_, turns out to be a poor classifier of the degree heterogeneity, while, unexpectedly, final epidemic size is a powerful predictor of network structure across the range of heterogeneity. We also evaluate our framework on empirical epidemiological data from past and recent outbreaks to demonstrate its application in practice and to gather insights about the relevance of particular contact structures for both specific systems and general classes of infectious disease. We thus introduce a simple approach that can shed light on the unobserved connectivity of a host population given epidemic data. Our study has the potential to inform future data-collection efforts and study design by driving our understanding of germane epidemic measures, and highlights a general inferential approach to learning about host contact structure in contemporary or historic populations of humans and animals.

## 1. Introduction

The accurate mathematical modelling of infectious disease outbreaks is important as a tool to understand and predict epidemic dynamics and evaluate the effectiveness of intervention strategies. In the context of directly transmitted pathogens, this ability relies, in part, on an understanding of the contact patterns between the individuals (hosts) of a population. Traditional epidemiological modelling accounts for contact behaviour through both implicit and explicit mechanisms and at different levels of abstraction. Purely compartmental models are used to model subsets of populations of individuals and allow different parameters to govern the rates of interaction between each. This approach has been successfully applied in capturing some of the heterogeneity inherent in contact patterns between groups of hosts. Contact network models, by contrast, explicitly define potential disease-transmitting connections between all individuals in a population, allowing for the incorporation of heterogeneous mixing at the lowest level. Contact networks represent individuals as network *nodes* and represent potentially disease-transmitting contact between individuals as network *edges*. The total number of edges connecting an individual with others is referred to as the individual's *degree*. The probability distribution of these degrees for all individuals in a network (i.e. population) is referred to as the network's *degree distribution*. The contact heterogeneity in a population is thus reflected in the variance of the network's degree distribution. Gaining an understanding of a host population's distribution of contacts provides structural information useful in characterizing and intervening in disease outbreaks, and has been demonstrated in numerous human, wildlife and livestock disease systems (e.g. [1–10]).

Gathering direct information about contact patterns via survey-based or device-based techniques to parametrize network models is often a time- and labour-intensive process, however [11,12]. Typically, these data are used to parametrize probabilistic contact network models. These models can then be used as the basis for predictive or public health intervention studies. While an understanding is beginning to emerge of which network structures are relevant for different classes of infectious disease, it is far from clear how to choose among different models of contact heterogeneity. That is, the probabilistic approach assumes a certain model *a priori* that may or may not be relevant to the disease of interest. (Alternatives to this paradigm do exist; notably, synthetic network models that use census and other socio-demographic data to infer possible contact networks using a first-principles approach [5,13].)

An alternative to these data-hungry strategies is a statistical approach, where a contact network model is inferred using available host and epidemic data within a likelihood framework [12]. A key benefit to this approach is that, in addition to providing information on which contact network model likely produced the observed data, the likelihood of alternative models can be evaluated to see how justifiable that choice is in relation to those alternatives. Previous work using a statistical approach has been carried out, where population structure is inferred using a broad range of data from infection/recovery times [14–17] to viral molecular sequence data [18,19]. However, much of this inferential work is specific to certain disease systems, is based on severe assumptions about the underlying contact structure or requires significant amounts of outbreak data [12]. A general framework that uses available data and informs future collection of epidemiological data is thus necessary.

In this study, we take an initial step towards this goal and evaluate the utility of commonly gathered epidemiological data for inferring contact network heterogeneity using a likelihood-based model selection framework. By using data that are commonly available for a variety of different disease systems, our approach seeks to make optimal use of collected data and inform future collection efforts. We evaluate three classes of network models that can be broadly classified by their increasing level of contact heterogeneity, looking specifically at whether it is possible to distinguish between these types using high-level and common epidemiological summary statistics. In this way, we are not so much attempting to pin down an exact network, but instead looking to determine what level of heterogeneity the data support, and which individual epidemiological metrics provide the most confidence towards this support. We test our inferential framework with synthetic and empirical contact data, comparing model selection results for each individual epidemiological measure with ground truth (i.e. the network class that generated it). We also evaluate our framework on empirical epidemiological data from historical and recent outbreaks, where the underlying contact structure is unknown, to demonstrate how our framework could be applied in practice and used to gather insights about the relevance of certain contact structures for general classes of infectious disease. In doing so, we determine that only certain epidemiological statistics are informative and consistent in recovering the level of contact heterogeneity in an underlying host population.

## 2. Material and methods

Here, we present our inferential framework in general, the contact network models and epidemiological data types that specify it as well our method for generating likelihood functions. In addition, we will also describe three procedures for testing the framework, using both synthetic and empirical data.

### 2.1. Bayesian classification of contact heterogeneity

We design a Bayesian classification or model selection framework [20–22] to infer contact structure heterogeneity in the host population based on commonly gathered summary measures in infectious disease epidemiology. For a given set of *m* contact network models, 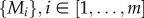, the posterior probability of each model given data, *X*, and the per-contact probability of transmission (referred to as transmissibility from here on), *T*, is2.1
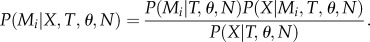


We specify this general framework by network models *M*_*i*_ which are parametrized by a population size, *N*, and a degree distribution with parameter *θ*. Thus, networks of class *M*_*i*_ are assumed to be simple and static random graphs of size *N* with specified degree distributions. While this is a simplifying assumption, networks of this class are well studied and are found to be suitable models of contact structure relevant to rapidly spreading epidemic diseases [5,23,24]. We select three (*m* = 3) random graph models (*M*_*i*_) of specified degree distributions with 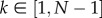:

—Poisson, with degree distribution2.2
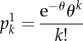


—‘exponential’^1^, with degree distribution2.3
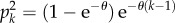


—scale-free, with degree distribution2.4
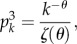
where *ζ* is the Riemann zeta function.

We choose these distributions as representatives of a spectrum of network structures, and to facilitate comparisons with previous work. The prior on the network models is taken to be uniform and independent of *T*, *θ* and *N*. If priors are available for any of these parameters, they can be included in this analysis. We choose to present the results over a range of fixed values of *T*, *θ*, *N* to discern any patterns in the classification based on these parameters.

Further, we specify *X*, the data, in equation (2.1) to individually be four common epidemiological metrics. These metrics capture the impact of an epidemic on public health systems: (i) *R*_0_, the basic reproductive ratio, which represents the initial growth rate of an outbreak and we calculate it empirically as the average number of secondary cases in the early part of the outbreak; (ii) epidemic peak size (*ρ*), which is the maximum number of infected individuals at any generation, and represents the maximum capacity surge on public health systems; (iii) epidemic size (*σ*), which is the total number of infected individuals, and represents the total burden on public health capacity; and (iv) epidemic duration (*δ*), which is the number of generations the outbreak lasts and represents the length of burden on public health systems.

The likelihoods, 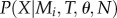 (for each *M*_*i*_ and *T* value, for fixed *θ* and *N*), can be acquired through either an analytical or a simulation approach. A simulation-based approach is used here to estimate the likelihood function for each data type (i.e. epidemiological measure). We use the configuration model [25] to generate instances of simply connected random graphs of size *N* and degree distribution as specified in equations (2.2)–(2.4). Subsequently, we perform Monte Carlo simulations for a susceptible–infected–recovered (SIR) epidemic model with a single initial infected case and per-contact transmissibility, *T*, on these networks, generating frequency distributions for each epidemic measure to use as likelihood functions. (This approach has similarities to the approximate Bayesian computation approach used for Bayesian inference [26–28].) The alternative approach would be to use an analytical epidemic model to generate a likelihood function for each epidemiological measure, based on the network class and pathogen transmissibility, and would eliminate the need for simulations. We make a preliminary evaluation of such an analytical framework [29] in the electronic supplementary material.

The classification of a population's contact heterogeneity occurs through model selection among the network models *M*_*i*_. Given one epidemiological datum, *X* (of type *R*_0_, *ρ*, *σ*, or *δ*), parameters *T*, *θ*, *N* and the likelihoods generated earlier, a posterior probability is calculated for each model *M*_*i*_ using equation (2.1). The selected network model is the one with the highest posterior probability.

### 2.2. Evaluating the classification framework

We test our framework in three stages using (i) synthetic contact network and synthetic epidemiological data to understand the inferential power of each epidemiological measure under idealized conditions; (ii) empirical contact network and synthetic epidemiological data to assess how informative each measure is given complex underlying network structure but idealized epidemiological assumptions; and (iii) empirical epidemiological data from historical and recent epidemic outbreaks where the contact network is unobserved to evaluate this method on systems with both complex network structure and complex epidemiology.

In each case, the provided epidemiological data (synthetic in the first two testing stages, empirical in the third) are summarized as the four metrics defined above (*R*_0_, *ρ*, *σ*, or *δ*), and used to calculate posterior probabilities of each network class, *M*_*i*_. In the first two testing stages, we have knowledge of the underlying contact network structure (synthetic for the first stage, empirical for the second), and will use it to judge the classification of contact heterogeneity as inferred by our method.

#### 2.2.1. Synthetic testing data

To systematically test our framework, we simulate epidemiological data on generated contact networks. Using the configuration model [25], we generate 10 random networks of size N=10 000 for each degree distribution specified in equations (2.2)–(2.4), where *θ* is chosen so that the mean degree is approximately 6 (±0.1). (We discuss the sensitivity of these results to network size and mean network degree in the electronic supplementary material.) We generate epidemic data on each of these networks with 10 000 SIR epidemic simulations, with transmissibilities, *T*, ranging from 0.1 to 0.5, at 0.05 intervals. The transmission probabilities are chosen to represent a range of pathogens, from low-transmissibility pathogens such as severe acute respiratory syndrome and influenza to more highly transmissible pathogens such as measles. Each of the epidemic simulations are then summarized as the four epidemiological measures, *R*_0_, *ρ*, *σ*, *δ*, to be used as data to test the classification framework. Classification of contact heterogeneities (i.e. network class model selection) based on this data occurs through the calculation of posterior probabilities, using equation (2.1), the likelihoods 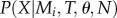 (for each *M*_*i*_ and *T* value, for fixed *θ* and *N*, as described by the synthetic data) and a uniform prior, 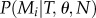.

#### 2.2.2. Empirical network testing data

In addition to systematic testing with synthetic epidemic data on simulated networks, we also test our framework with synthetic epidemic data on empirical contact networks from various studies spanning human and livestock systems. The purpose of these experiments is to control for complexities in epidemiology by confronting our framework with empirical contact network data that do not conform to the structural assumptions made in our synthetic networks (above). The number of nodes, *N*, is provided in each study, and the transmission probability, *T*, is chosen so that an epidemic resulted in an expected final size of approximately 0.25*N*. The four chosen datasets are the following.

— A contact network (for a sexually transmitted disease) based on surveys of romantic and sexual relationships between adolescents in a mid-size Midwestern US town [2] (*N* = 278, *T* = 0.75). (We refer to this network as ‘adolescent sexual’.)
— A high-resolution network of cattle movement between farms in the UK during the month of April 2004 [30] (*N* = 37 787 *T* = 0.22). (We refer to this as ‘cattle’.)
— A high-resolution radio-frequency identification (RFID) tag-based network of face-to-face interactions lasting at least 10 min among students and staff at a California high school [31] (*N* = 661, *T* = 0.27). (We refer to this as ‘school’.)
— An urban contact network (for a respiratory disease) generated based on data from Vancouver, British Columbia [5,10] (*N* = 12 729, *T* = 0.072). (We refer to this as ‘urban’.)

(Further information on network topology for each dataset is provided in the electronic supplementary material.)

We simulate 1000 epidemics on these empirical contact networks to produce replicate data on epidemiological measures, *R*_0_, *ρ*, *σ*, *δ*. Classification of contact heterogeneities (i.e. network class model selection) based on these data occurs through the calculation of posterior probabilities, using equation (2.1), the likelihoods 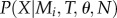 (for each *M*_*i*_ and *T* value, for fixed *θ* and *N*, as defined in the empirical study) and a uniform prior, 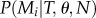.

For validation of the contact structure inferred by these epidemiological data, we statistically fit the degree distributions of the known empirical networks. For each of our datasets, we evaluate three one-parameter candidate distributions (Poisson, exponential, scale-free) using maximum-likelihood estimation (MLE) to fit the distribution parameters. We then use the Kullback–Liebler divergence (K–L divergence) to select the most appropriate degree distribution for the data. The best-fit distribution for each dataset is presented in §3, and is used for validation of our inferred results (see the electronic supplementary material for more details).

#### 2.2.3. Empirical epidemic testing data

Lastly, we test our framework with data from empirical epidemic outbreaks. These data come from studies that do not necessarily provide an estimate of the underlying contact network, but do provide an estimate of at least one epidemiological measure: epidemic final size, peak size or duration. In every case, an estimate of *N* and either an estimate of the mean degree or *R*_0_ were provided. In cases where an estimate of the mean degree was provided, it was used to determine a fixed value of *θ* in equations (2.2)–(2.4) so that each had a mean degree value matching the estimate. In cases where an estimate of mean degree is not provided, we assume a broad range of values for mean degree, and use a range of values for *R*_0_ (provided in the study or from the literature), to calculate a value for *T* based on the following relationship (where *p*_*k*_ is the proportion of nodes with degree *k* from equations (2.2), (2.3) or (2.4)) [5]:
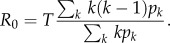


The three empirical outbreaks are results of respiratory, sexual and food-borne diseases, respectively.

— *Measles:* a severe outbreak of measles in 1861 in the isolated village of Hagelloch, Germany resulted in all children under the age of 14 being infected [15,32,33]. The susceptible population (children of age < 14 who did not have maternal immunity) was of size *N* = 185, and the epidemic produced a final size of 185 individuals. The value of *R*_0_ was assumed to be between 6 and 10 [34], and the mean degree was varied from 8 to 30.
— *Gonorrhea:* in early 1999, a localized outbreak of *Neisseria gonorrhea* occurred in Alberta, Canada [35]. The susceptible population (for which the data were collected) was *N* = 39, of which 20 individuals were infected. A mean number of contacts per individual (mean degree) of 2.1 was measured in the study, and *R*_0_ was assumed to be between 1 and 3 [36].
— *Norovirus:* in the summer of 2004, there was a norovirus outbreak at an international scout jamboree in The Netherlands, which was divided into seven camps [37]. We use data provided on two of the camps that became infected: (i) camp ‘1’: *N* = 721, epidemic final size = 77, peak size = 19 and (ii) camp ‘2’: *N* = 825, epidemic final size = 41, peak size = 16. For the inference, we make the simplifying assumption that each camp did not mix with others and thus treat them as isolated epidemics. *R*_0_ was assumed to be between 1.88 and 2.3 as estimated in the study, and mean degree is varied from 5 to 20.

Classification of contact heterogeneities (i.e. network class model selection) based on the epidemiological datum provided in each study occurs through the calculation of posterior probabilities, using equation (2.1), the likelihoods 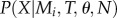 (for each *M*_*i*_ and *T* value, for fixed *θ* and *N*, as specified in the empirical study or inferred) and a uniform prior, 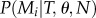. We note that these experiments are performed for a range of values of the mean degree or *T* so as to characterize the dependence of the network inference to variation in these parameters (which does exist naturally).

## 3. Results

Using the Bayesian model selection framework described earlier, we evaluate the inference of contact heterogeneity using four common epidemiological metrics: *R*_0_, the reproductive ratio; *ρ*, the epidemic peak size; *δ*, the epidemic duration; and *σ*, the epidemic final size. Given each data type, we consider the posterior probabilities of three network classes (random graphs with degree distributions of Poisson, exponential and scale-free), representing the range of degree heterogeneity (from the fairly homogeneous Poisson to the highly heterogeneous scale-free). We emphasize that we are not attempting here to pin down an exact network, but instead are looking to determine what level of contact heterogeneity is supported by the data, and which individual epidemiological metrics provide the most confidence towards this support.

### 3.1. Testing with synthetic network data

Here, we assess the inferential strengths and limitations of epidemiological data to infer underlying contact heterogeneity, using synthetic epidemic data generated on synthetic host population contact networks. The four epidemiological measures (*R*_0_, *ρ*, *σ* and *δ*) gathered from the synthetic epidemic data display different utilities as classifiers among themselves and, notably, among different transmission probability values.

**Figure 1.**
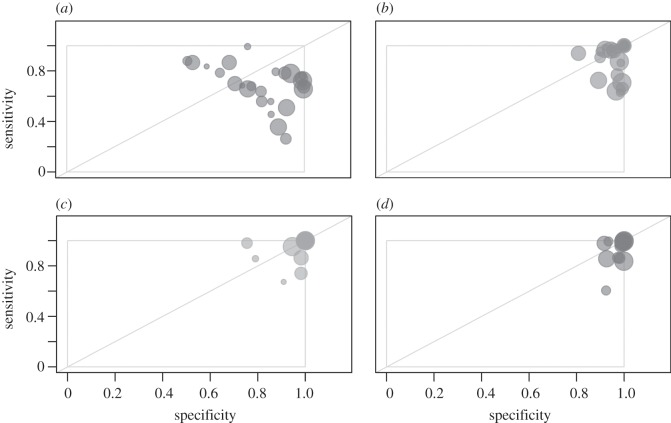
Reliability of classifier based on synthetic data experiments: (*a*) *R*_0_, (*b*) peak size (*ρ*), (*c*) final size (*σ*) and (*d*) duration (*δ*). The plot shows the reliability of each epidemic metric-based classifier as measured by sensitivity, the true positive rate, and specificity, the true negative rate. Each point on the plot represents a network class and a transmission probability, and the relative size of each point indicates the transmission probability (larger points indicate higher transmission probabilities). All four classifiers have the same number of points plotted, although many do overlap.

To evaluate the reliability of the classifiers, we treat the results of the Bayesian analysis as a binary classification: for every network class, *M*_*i*_, we define a positive result as one where the model *M*_*i*_ is supported most (i.e. with the highest posterior probability 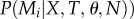 by the epidemic datum. Using these results, classified as positive and negative for each network class, we consider the sensitivity and the specificity of the epidemiological measures as classifiers. Sensitivity indicates the proportion of positives for which the true network class is inferred as most likely (true positive rate), while specificity indicates the proportion of negatives for which the network classes that are not true are not inferred as most likely (true negative rate; more information is provided in the electronic supplementary material). Together, these two ratios present a picture of how reliable each measure is at classifying the networks. The results shown in figure 1 highlight that overall the classifiers perform well, with only *R*_0_ having a trade-off between specificity and sensitivity, and tending to be more specific than sensitive. Epidemic final size, peak size and duration, on the other hand, tend to balance both high specificity and sensitivity (for most transmission probabilities). While peak size and duration perform better as transmissibility decreases, final size classifies more effectively for increasing transmissibility values. However, sensitivity and specificity only account for categorical model choice and do not take into account model selection uncertainty.

**Figure 2.**
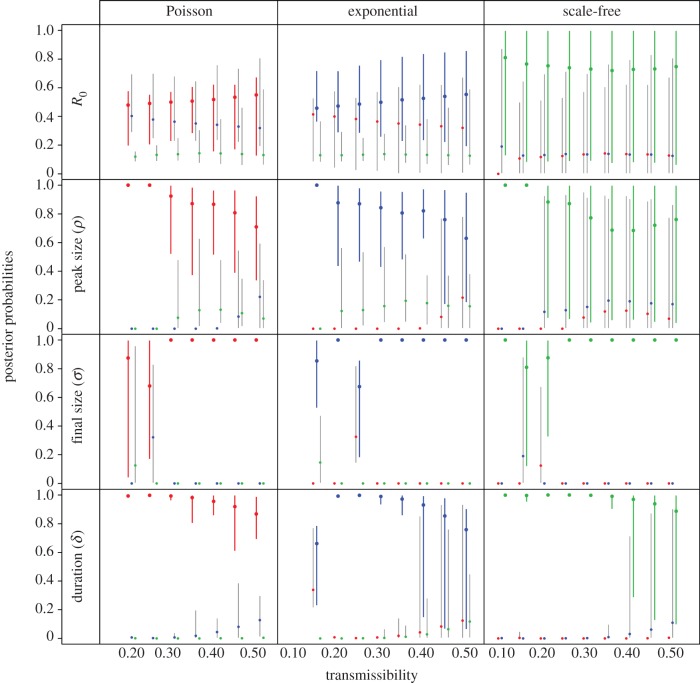
Bayesian classification predictions for synthetic data. Posterior probability values, *P*(*M*_*i*_|*X*, *T*, *θ*, *N*), are shown on the *y*-axis for all synthetic data classified, separated by the underlying network type (Poisson, exponential, scale-free), epidemiological metric (*R*_0_, *ρ*, *σ*, and *δ*) and transmission probabilities (from *T* = 0.05 to *T* = 0.5). Boxes are coloured in each cell to highlight the posterior values of the network type that actually generated the data: Poisson (red), exponential (blue) and scale-free (green), and dots indicate the median of each set of posterior probabilities. Boxes represent the 95% equal-tail credible intervals and are only shown when the number of samples used to determine them was greater than 1000.

An evaluation of the posterior probabilities provides a more quantitative measure of the confidence in each network type given the data. These probabilities are shown for each epidemiological measure (across all network classes and transmission probabilities) in figure 2 and describe the utility of the measures as classifiers. Predictions based on *R*_0_ are reliable for the most heterogeneous (scale-free) networks (albeit with large variances) across transmission probabilities, but are largely ambiguous for Poisson and exponentially distributed networks. Classifications based on *ρ* and *δ* become less effective for higher *T* values; that is, sufficiently transmissible pathogens propagate through all population types efficiently, leading to similar-sized epidemic peaks and durations. Contact heterogeneity based on *σ*, final size, is most consistent across *T* values and thus is appropriate to use when transmissibility is not well known *a priori*. Although the certainty in classification decreases for moderate values of *T* (0.15–0.25) (see the electronic supplementary material, figure S4, for comparison of final size distributions), the classification is still strong. The *σ*-classifier also remains most effective (for all *T* values) for populations with smaller (<2) or larger mean degrees, or for smaller population sizes (see sensitivity analysis in the electronic supplementary material). Overall, these results show that all epidemic measures are most sensitive towards scale-free networks and least towards exponentially distributed networks.

### 3.2. Testing with empirical network data

**Figure 3.**
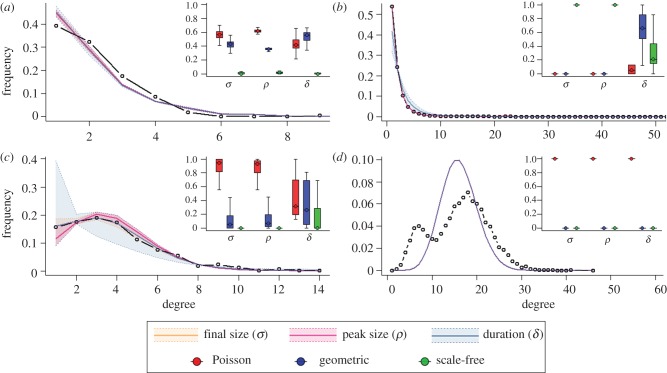
Bayesian classification results on empirical network data: (*a*) adolescent sexual, (*b*) cattle, (*c*) school and (*d*) urban. Actual degree distributions (black with points) are shown against the posterior degree distributions generated for each of the three epidemiological measures used (final size (*σ*), peak size (*ρ*) and duration (*δ*)) and are coloured according to the legend, where the thick line indicates the median and thin dotted lines represent the upper and lower 95% equal-tail credible intervals. The actual posterior values used are also shown (insets) and are broken down by measure (symbols) and network class (colour).

We now use synthetic epidemiological data generated on four empirical contact network populations to assess how informative each measure is given complex underlying network structure but idealized epidemiological assumptions. Our results are validated in each case by knowledge of the true contact network and its statistical best fit. In figure 3 (box plots) we show, for each empirical contact network (adolescent sexual, cattle, school and urban), the posterior probabilities of each network class based on three of the four data types, *σ* : final size, *ρ* : peak size, *δ* : duration. (We choose to ignore *R*_0_ in this analysis, given its poor performance in the previous testing stage.) In addition, for each empirical contact network, we show the *posterior degree distribution*, calculated via Bayesian model averaging [20,38],
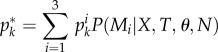
as the sum of the three model degree distributions weighted by the posterior probabilities for each network class, *M*_*i*_. For each network class *i*, the model degree distributions, *p*_*k*_^*i*^, are generated using equations (2.2)–(2.4), with *θ* fitted to each empirical contact network dataset.

The degree distribution of the adolescent sexual network (figure 3*a*) has a best fit of exponential (using the K–L divergence values, given the MLE estimates). (All K–L divergence values and best-fit distributions are shown in the electronic supplementary material, table S1, and figures S9–S12.) It is inferred by our model selection as Poisson based on the final size (*σ*) and peak size (*ρ*) classifiers, but as exponential by the duration (*δ*) classifier. This result confirms our sensitivity analysis that all classifiers for networks of mean degree two, for small population sizes, are relatively ambiguous for the transmission probability used here (see sensitivity analysis in the electronic supplementary material). For the cattle movement network (figure 3*b*), a scale-free network is selected strongly by statistical fitting, and both the *σ* and *ρ* classifiers strongly prefer the same. The *δ* classifier provides significant evidence for the exponential network, reflecting the disassortative nature of this network [39]. For the school network (figure 3*c*), the Poisson degree distribution is supported by both our model selection and by the K–L divergence best fit. The *δ* classifier does provide weaker support, due to the small population size (see sensitivity analysis in the electronic supplementary material). Lastly, the epidemiological data produced over the urban network (figure 3*d*) strongly match the K–L divergence best fit of a Poisson degree distribution. The true degree distribution of this empirical network looks to be more complex than any of the three network classes, but its dominant feature, a dispersed peak with a moderate average degree, is most consistent with a Poisson distribution. In addition, the network is strongly modular and moderately assortative (see the electronic supplementary material), but still acts like a Poisson-distributed random network with respect to epidemic outcomes.

These results underline that the common epidemiological measures we have chosen to use as data within the Bayesian model selection framework can be useful at correctly classifying the level of contact heterogeneity in a population. Epidemic final sizes are most consistent in this respect, except in the case of small population sizes with extremely low connectivity, which is in accordance with our results using the synthetic testing data. Epidemic duration, although effective when population size and mean degree are small, is not strongly reliable otherwise (see the electronic supplementary material, figure S7). These results demonstrate that although each of these networks has complex network structure (clustering, assortativity, modularity), a classification framework based on the assumption of randomness still predicts the contact heterogeneity as reflected in the degree distribution well. However, they also suggest that network features (such as significant disassortativity) that are a result of more than the degree distribution are not captured by these epidemic measures (especially duration). In addition, these findings highlight that the framework works well across a broad range of mean degree values (2–16) and transmission probabilities (0.07–0.75).

### 3.3. Classification of empirical epidemic data

**Figure 4.**
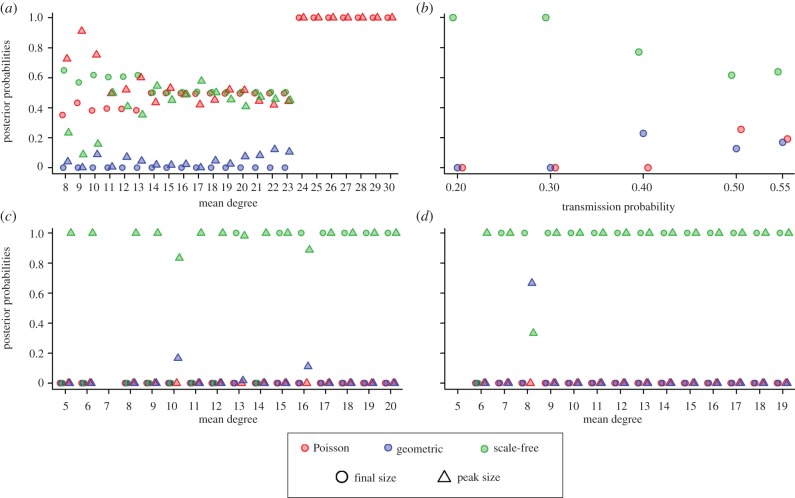
Bayesian classification on empirical epidemic data: (*a*) measles, (*b*) gonorrhea, (*c*) norovirus, camp 1 and (*d*) norovirus, camp 2. Posterior probabilities are shown for each set of empirical outbreak data: measles, gonorrhea and norovirus within the two camps. Results are broken down by network class (colour) and epidemiological measure (shape). The *x*-axis shows a range of either transmission probabilities (*T*) or mean degree (*θ*) values depending on which was unknown for each study.

The results of the previous two sections elucidate how informative various epidemic measures are about population contact heterogeneity, even in the presence of complex network structure. Given these results, we now infer contact heterogeneity under the three models using epidemic data from some historical and recent outbreaks where the population contact structure is unknown, with our results presented in figure 4.

For a measles outbreak from the small town of Haggelloch, Germany (figure 4*a*), the final size and peak size posterior likelihoods for each network class and various mean degrees suggest that the Poisson contact network is the most likely model, albeit not strongly so, except where the mean degree value is greater than 23. The choice of Poisson is reasonable in that it has the lowest contact heterogeneity of the three contact network models and measles has been relatively well modelled in the past using a homogeneous-mixing model [40,41]. The scale-free network model also has relatively high posterior probabilities at lower mean degree values. The most likely explanation for this result is that the mean degree among children less than the age of 14 in Hagelloch was in fact greater than 23 (where the Poisson model is the only likely model). This hypothesis is not unreasonable as the school class sizes in Hagelloch varied from 30 to 90 children. (However, this result is at odds with the results of Groendyke *et al*. [15,42] who predict an average degree of 8–12.)

For a gonorrhea outbreak from a town in Alberta, Canada (figure 4*b*), for transmission probabilities in the range 0.1–0.55, the scale-free model is strongly preferred. At higher transmission probabilities (0.6+, not shown), Poisson and exponential network models are more likely. Empirical evidences suggest that gonorrhea has a transmission probability lower than 0.55 due to characteristics of the bacterium itself [43,44]. Research also suggests that the scale-free model is a reasonable prediction in this case, as degree distributions of human sexual contact networks have been shown to exhibit high levels of contact heterogeneity and are characterized by a core group of highly active individuals that tend to bridge more isolated individuals [45–48].

For a norovirus outbreak from two camps in a children's summer jamboree (figure 4*c*,*d*), the epidemic final and peak sizes strongly indicate that a scale-free network underlies the population in both cases. Norovirus typically spreads very quickly in a population, usually by person-to-person contact by means of faecal–oral or aerosol transmission [37]. Multiple factors mediate norovirus transmission such as host movement and environmental contamination and thus what constitutes a potentially disease-transmitting contact is vague. It is unclear in this study which factor drove the outbreak, but our data support an underlying population with a large degree of heterogeneity in their disease-causing contacts. This heterogeneity could point to variation in individual hygiene behaviour or be the result of primary environmental transmission. The model selection results are consistent across all mean degree values, thus adding more confidence to the prediction.

For all three of these studies, the results of our model selection framework are mainly consistent with known attributes of each disease. Overall, the framework based on epidemic size and/or peak size gives an accurate characterization of the support for various levels of contact heterogeneity and therefore gives important information towards future model development for each disease system. In addition, all predictions are fairly consistent across the range of mean degrees or transmission probabilities. Although we are not explicitly estimating other parameters for any of these systems, the model selection for network class can be followed up by model selection on the mean degree (or more precisely, the value of *θ*) and the transmission probability [20].

## 4. Discussion

Directly transmitted pathogen dynamics are fundamentally driven by the interactions between individuals in the host population that make up infectious contact and lead to transmission. Network epidemiology has come a long way in demonstrating the impact that the structure of these interactions (which make up the contact network) has on the progression of an infectious disease [5,11,24,49–52]. In this study, we have developed a predictive framework to show that common epidemiological measures can give important insights into the contact structure of a host population. The important dependence that has been established between contact network structure and infectious disease dynamics is both a motivation for why a framework like ours is needed and a key to its development. In particular, we have focused on the heterogeneity in the host population's contact structure as represented through the degree distribution. Although degree heterogeneity is only one of the characteristics that describes a network, it has been understood to play a fundamental role in describing variation in disease transmission [24,53], and has been recently shown to in fact account for much of the variation in many cases [54].

From our analysis, it is evident that some epidemiological measures better distinguish contact heterogeneities in the underlying populations. The reproductive number does seem to have some potential as a classifier, but performs poorly, especially for less-connected populations. (In our sensitivity analysis, shown in the electronic supplementary material, we find that the *R*_0_ classifier improves for more connected populations (mean degree ≥ 10).) All the remaining measures also have mixed results for varying parts of the mean degree and transmissibility parameter space. (Given these results, the development of a joint likelihood approach that combines information from multiple epidemic measures could be productive. Although not the goal of this study, we present a preliminary analysis based on combined metrics in the electronic supplementary material.) Overall, our primary and sensitivity analyses indicate that a less-connected host population with a moderate or highly transmissible pathogen (as often occurs in human sexually transmitted disease systems) can best be classified by final size or peak size; while a highly connected host population with a low or moderately transmissible pathogen (as often is the case for human respiratory disease outbreaks) is better classified by final size and in many cases by duration. That final size is a good classifier is likely due to the low variability it exhibits for a given contact network structure. The exception to the effectiveness of final size as the most reliable metric is that of an extremely sparsely connected (mean degree ≈ 2) and small host population sizes (*N* < 500).

The inferential results from epidemiological data generated over known empirical contact networks, each constructed via different methods, show that our model selection framework is robust in the face of contact networks that do not strictly conform to one of the three model classes. The fact that the model selection is accurately able to classify the contact structure (from the three model options) reconfirms that the degree distribution characterizes the network structure well for epidemic outcomes, even in the presence of complex network characteristics such as transitivity and assortativity. (Further work is needed, however, to detect other secondary structures such as modularity from epidemiological data.) In addition, the posterior degree distributions specify reasonable approximations to the true degree distributions, providing a more informed *a priori* contact structure for predictive and intervention modelling studies of future outbreaks.

When applied to epidemiological data from populations where little is known about the underlying contact network, our framework provides important insights into the structure of these populations and illustrates how this method can be used in practice. In contrast to the sample sizes in our previous experiments, these predictions are based on one data point each, and still produce predictions that are congruent with our understanding of the respective disease systems. These predictions can also begin to shed some light on the relationship between network structure and certain classes of disease systems (i.e. a pathogen with a given transmission mode and the type of population it spreads in). Sexually transmitted disease spread in heterosexual and homosexual populations has been the most well-studied case in this regard. There has been a significant amount of work investigating the high degree of heterogeneity found in sexual contact networks, made up of the monogamous many and promiscuous few [45,46,48]. The spread of childhood diseases such as measles spreading among cohorts of susceptible children is also a classic example where the level of heterogeneity has typically been assumed (to be low, in this case), although never shown. Our predictions on the Hagelloch outbreak provide evidence in this direction. Few other disease systems have been investigated in this manner and thus we have a poor understanding of whether or not certain contact structures characterize certain classes of diseases. The strong support towards high contact heterogeneity in the norovirus outbreaks points to transmission of gastrointestinal pathogens as being driven by super-spreading events, either due to strong environmental seeding or as a result of individual behaviour.

This initial framework does have notable limitations, however. Primarily, we have made assumptions on the pathogen spread that may not hold in all cases. We assume no incubation period, a constant generation time and no variability in susceptibility or transmissibility. The framework also assumes a static contact network, not accounting for dynamics in contact structure due to social changes or public health interventions [55]. Although these additional complexities are not expected to fundamentally weaken the approach, further work is necessary. Lastly, our approach is designed for large-scale epidemics only (i.e. *R*_0_ > 1 and the outbreak spreads to a significant proportion of the population). An important future challenge will be to develop a framework based on endemic or early outbreak surveillance data, which could be used to classify population structure before an epidemic occurs, so that the results could be used to predict and design interventions in real time.

Thus, we have designed a simple and general predictive framework that can shed light on a host population's connectivity, given epidemic data. Our approach has a low demand for data in terms of both quantity and availability, and has the potential to inform future epidemiological data-collection efforts and study design by driving our understanding of germane epidemic measures in the context of contact structure inference. While our study is a first step towards a larger goal, a statistical approach powered by existing (epidemiological) data could greatly contribute towards the characterization of host contact structure, without the need to spend resources or compromise privacy in accessible populations, while making it possible to describe unreachable human and animal populations of the past.

## Endnote

1 The ‘exponential’ degree distribution is a discrete probability distribution common in network theory.
